# The early Aurignacian dispersal of modern humans into westernmost Eurasia

**DOI:** 10.1073/pnas.2016062117

**Published:** 2020-09-28

**Authors:** Jonathan A. Haws, Michael M. Benedetti, Sahra Talamo, Nuno Bicho, João Cascalheira, M. Grace Ellis, Milena M. Carvalho, Lukas Friedl, Telmo Pereira, Brandon K. Zinsious

**Affiliations:** ^a^Department of Anthropology, University of Louisville, Louisville, KY 40292;; ^b^Interdisciplinary Center for Archaeology and Evolution of Human Behaviour (ICArEHB), Universidade do Algarve, 8005-139 Faro, Portugal;; ^c^Department of Earth and Ocean Sciences, University of North Carolina Wilmington, Wilmington, NC 28403;; ^d^Department of Human Evolution, Max Planck Institute for Evolutionary Anthropology, 04103 Leipzig, Germany;; ^e^Department of Chemistry, University of Bologna, 40126 Bologna, Italy;; ^f^Department of Anthropology and Geography, Colorado State University, Fort Collins, CO 80521;; ^g^Department of Anthropology, University of New Mexico, Albuquerque, NM 871317;; ^h^Department of Anthropology, University of West Bohemia, 30614 Plzen, Czech Republic;; ^i^Department of History, Arts, and Humanities, Universidade Autónoma de Lisboa, 1169-023, Lisbon, Portugal;; ^j^Centro de Arqueologia da Universidade de Lisboa (UNIARQ), 1600-214, Lisbon, Portugal;; ^k^Centro de Geociências, Universidade de Coimbra, 3030-790, Coimbra, Portugal;; ^l^Department of Anthropology, University of Connecticut, Storrs, CT 06269

**Keywords:** Aurignacian, modern human, dispersal, Iberia, Paleolithic

## Abstract

Documenting the first appearance of modern humans in a given region is key to understanding the dispersal process and the replacement or assimilation of indigenous human populations such as the Neanderthals. The Iberian Peninsula was the last refuge of Neanderthal populations as modern humans advanced across Eurasia. Here we present evidence of an early Aurignacian occupation at Lapa do Picareiro in central Portugal. Diagnostic artifacts were found in a sealed stratigraphic layer dated 41.1 to 38.1 ka cal BP, documenting a modern human presence on the western margin of Iberia ∼5,000 years earlier than previously known. The data indicate a rapid modern human dispersal across southern Europe, reaching the westernmost edge where Neanderthals were thought to persist. The results support the notion of a mosaic process of modern human dispersal and replacement of indigenous Neanderthal populations.

## Significance

We report the remarkable discovery of an early Aurignacian occupation, ∼5,000 years older than any Upper Paleolithic site in westernmost Eurasia. The archaeological and radiocarbon data provide definitive evidence that modern humans were in western Iberia at a time when, if present at all, Neanderthal populations would have been extremely sparse. This discovery has important ramifications for our understanding of the process of modern human dispersal and replacement of Neanderthal populations. The results support a very rapid, unimpeded dispersal of modern humans across western Eurasia and support the notion that climate and environmental change played a significant role in this process.

**Fig. 1.**
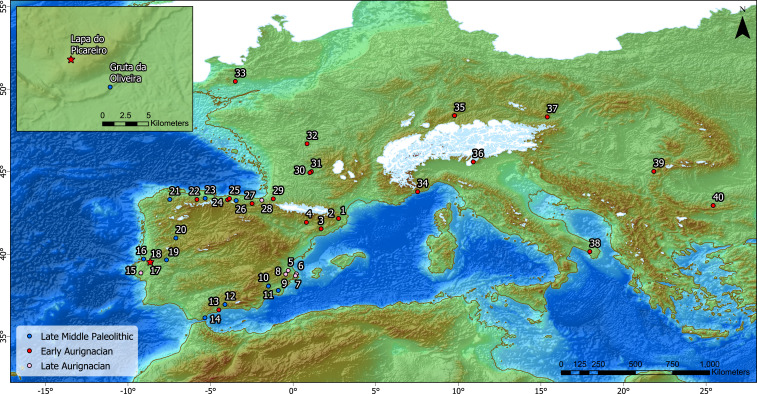
Map of selected Early Upper Paleolithic (red and pink) and Late Middle Paleolithic (blue) sites in Europe (7). (1) Arbreda, (2) Reclau Viver, (3) Abric Romaní, (4) Cova Gran, (5) Mallaetes, (6) Cova Foradada, (7) Cova de les Cendres, (8) Sima de las Palomas, (9) Cova Beneito, (10) La Boja, (11) Cueva Antón, (12) Zafarraya, (13) Bajondillo, (14) Gorham’s Cave, (15) Pego do Diabo, (16) Mira Nascente, (17) Lapa do Picareiro, (18) Gruta da Oliveira, (19) Foz do Enxarrique, (20) Cardina, (21) A Valiña, (22) La Viña, (23) El Sidrón, (24) El Castillo, (25) Cueva Morín, (26) El Mirón, (27) Labeko Koba, (28) Aitzbitarte III, (29) Isturitz, (30) Abri Castanet, (31), Abri Pataud, (32) Les Cottés, (33) Kent’s Cavern, (34) Riparo Mochi, (35) Geissenklosterle, (36) Fumane, (37) Willendorf, (38) Grotta del Cavallo, (39) Pestera cu Oase, and (40) Bacho Kiro.

The dispersal of modern humans across western Eurasia, associated with the Upper Paleolithic, is well documented compared to other regions but still susceptible to discoveries that can overturn prevailing ideas, especially those based on first appearance dates (1, 2). Current data support an east-west dispersal beginning ∼46 ka cal BP in the Balkan Peninsula at Bacho Kiro (3, 4) (Fig. 1). Subsequently, modern humans spread up the Danube river basin and along the Mediterranean rim within a relatively short period (5). The process was likely a mosaic involving dispersal into empty spaces and interaction with indigenous Neanderthal populations. At some point, around 43 to 42 ka cal BP, the regional variants of the Initial Upper Paleolithic coalesced into the Aurignacian technocomplex, appearing synchronously across western Eurasia (6).

The Iberian Peninsula holds a peculiar place in the problem of modern human dispersal ever since the publication of unexpectedly early dates for the first Upper Paleolithic appearance at El Castillo, l’Arbreda, and Abric Romaní in northern Spain (8, 9). Subsequent dating from these and additional sites has constrained the Aurignacian arrival in the region to ∼43.3 to 40.5 ka cal BP (10–14). These dates verify a rapid modern human dispersal and allow for a temporal overlap of ∼1,000 years with Neanderthals in northern Spain (11), and longer in southern Iberia. Furthermore, the scenario is complicated by the lack of associated fossil remains, leaving open the possibility that either human group created the early Aurignacian assemblages in the Franco-Cantabrian region (15). Despite this lack of direct association for the early Aurignacian, modern human remains have been identified in this time frame in Romania (16) and Italy (17, 18). Since no Neanderthal sites contain evidence for the use of carinated technology to produce bladelets, we can assume that modern humans were the makers of the entire Aurignacian cultural complex.

Undeterred by this uncertainty, the early appearance dates for the Upper Paleolithic and late appearance dates for Middle Paleolithic Neanderthals in southern Iberia led to the construction of various models to explain the apparent biogeographic boundary separating the two populations (19, 20). In these models, ecological adaptations allowed Neanderthals to survive, preventing modern human dispersal in southern Iberia until ∼37 to 30 ka cal BP, a period of 6,000 to 12,000 years (21, 22).

The recent dating of Bajondillo cave, on the southern coast of Spain, arguably demonstrated the first presence of modern humans at ∼45 to 43 ka cal BP, suggesting an even more expansive dispersal across Europe in a geological blink of an eye (23). The new Bajondillo dates pushed the first appearance of modern humans several thousand years earlier in time, upsetting previously held views. Critics dismissed the dated lithic assemblage in level Bj13 as a mixed collection of artifacts from younger and older occupations or as lacking typological traits of the Proto or Early Aurignacian technocomplexes (24, 25). Until the Bajondillo publication, the earliest Upper Paleolithic held at ∼35 ka cal BP at Cova de les Cendres (Mediterranean coast) (26), ∼36.5 ka cal BP, at La Boja (22), (southern Spain), and ∼34.5 ka cal BP at Pego do Diabo, (central Portugal) (27), all attributed to the Evolved/Late Aurignacian.

On the flip side of the problem, the last appearance of Neanderthals and the Middle Paleolithic has also been cast in doubt (28, 29). The number of “late” Neanderthal sites (<42 ka cal BP) has diminished substantially since the application of new dating techniques showed them to be much older (>42 ka cal BP) than previously thought (30–33). Across central Spain, there are no Neanderthal remains or Middle Paleolithic sites dated after 42 ka cal BP (34–36). At present, Gruta da Oliveira, Cueva Antón, and Gorham’s Cave remain the only “late” Neanderthal sites in southern Iberia dated ∼37 ka cal BP or later (21, 22).

**Fig. 2.**
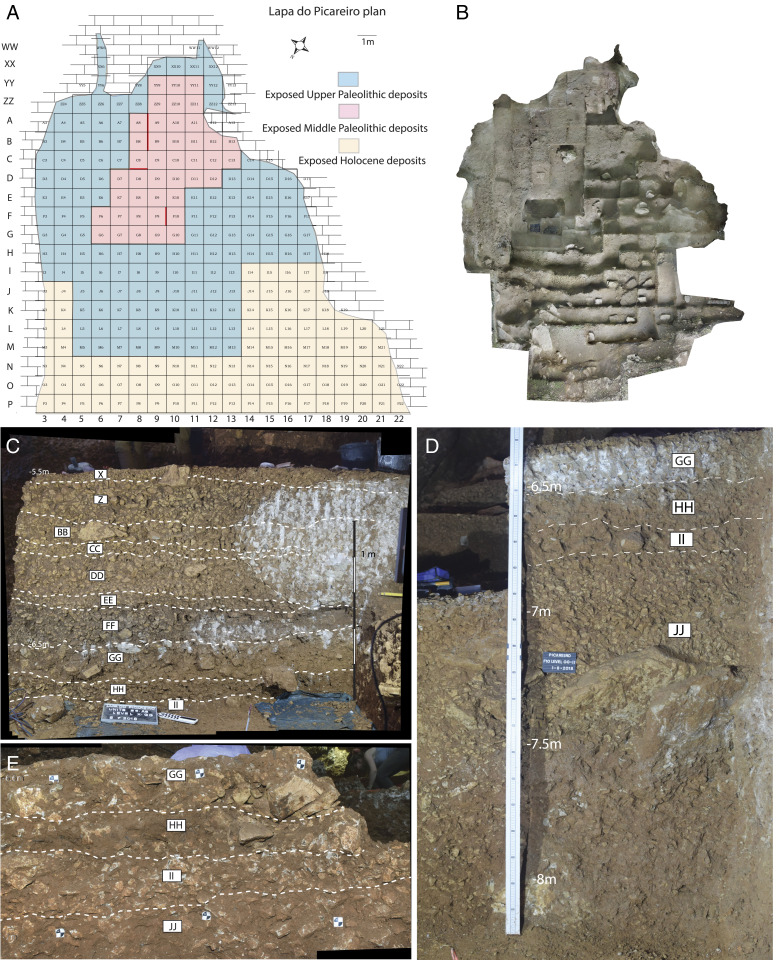
(*A*) Site plan with profile locations shown in red. (*B*) Orthophoto of the excavation from above. (*C*) Stratigraphic profiles for the MP-UP sequence in Lapa do Picareiro. (*D*) Level X to level II profile in units B8-A8, (*B*) level GG to level JJ in unit F9, (*E*) close-up of level GG-II in unit C8.

Clearly, sites dated ∼42 to 37 ka cal BP are extremely rare, suggesting that most of Iberia south of the Ebro basin was a sparsely, if at all, populated landscape into which modern humans could have easily dispersed. The lack of archaeological and fossil evidence for this interval could also be due to climatic and landscape instability that erased the record or prevented its formation (37, 38). Under these conditions, survivorship is likeliest in sedimentary traps or sheltered locations where accumulative processes preserve material evidence. One such place is Lapa do Picareiro, a limestone cave located 570 m above sea level, on the west-facing slope of Serra de Aire, a karst mountain north of the Tagus River valley in west-central Portugal (Fig. 1). The 15 × 15 m cavern (Fig. 2 *A* and *B* and *SI Appendix*, Fig. S1) is part of a large (25 × 30 m) bedrock depression with a thick sedimentary fill of muddy éboulis representing much of the Late Pleistocene. We have excavated a 10.6-m deep section with 36 Pleistocene-aged strata (levels E-NN), revealing a thick Upper Paleolithic sequence (levels E-II) continuing into the Middle Paleolithic (levels JJ-NN) (39, 40). Age determination of the levels comes from 80 radiocarbon dates produced over the last 25 y of investigation at the cave. The stratigraphic sequence in Picareiro has roughly 2 m of sediment dated ∼45 to 35 ka cal BP, corresponding to the temporal range of the Middle-Upper Paleolithic transition in southern Iberia (Fig. 2 *C*–*E* and *SI Appendix*, Figs. S2 and S3). As the ongoing excavation has exposed the deeper deposits in the back of the cave, evidence for previously undetected Early Upper Paleolithic occupations has emerged.

## Results

A series of dates using the ultrafiltration and enhanced collagen extraction pretreatments on anthropically modified ungulate bones from the Late Middle and Early Upper Paleolithic levels is presented here (*SI Appendix*, Table S1 and Figs. S4–S6). The results confirm an early Aurignacian presence in the region, potentially overlapping with level Bj13 from Bajondillo and positing significant implications for our understanding of modern human dispersal and late survival of Neanderthals in Europe.

**Fig. 3.**
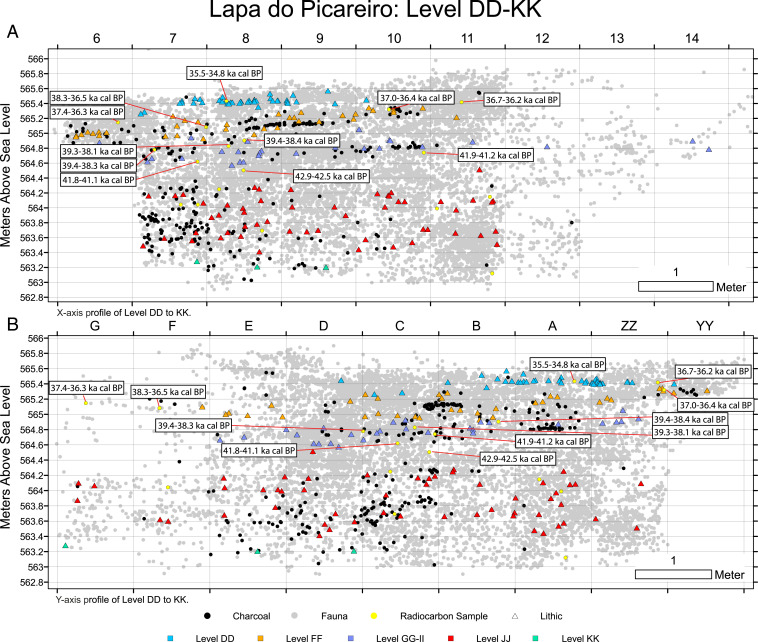
Plots of lithic artifacts along with radiocarbon-dated bone samples from Lapa do Picareiro: (*A*) X-axis profile of the cave, perpendicular to the central axis, (*B*) Y-axis profile of the cave, lengthwise from front to back.

**Fig. 4.**
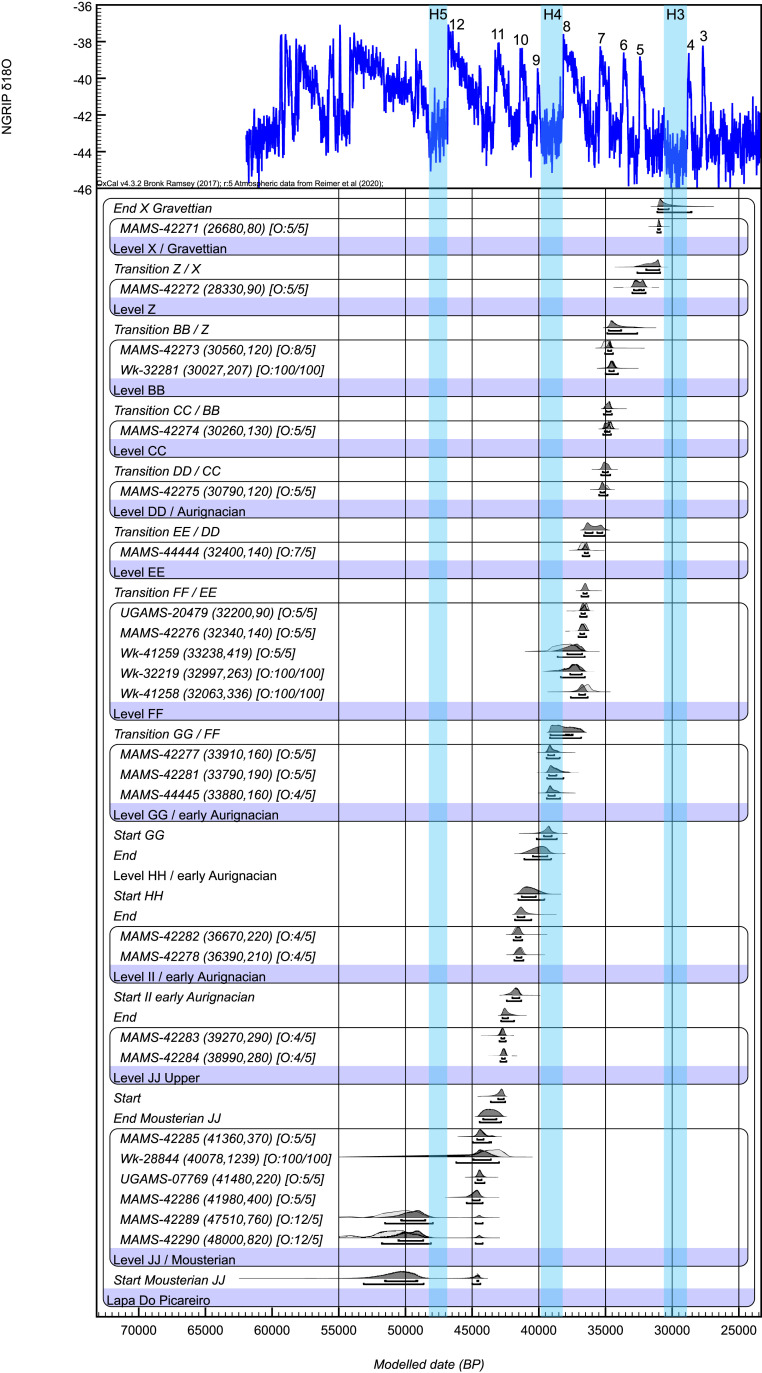
Bayesian model for the Lapa do Picareiro. Radiocarbon dates are calibrated using IntCal20 (41); the model and boundaries were calculated using OxCal 4.3 (42), including a general t-type outlier model. Outlier prior and posterior probability are shown in square brackets. Four samples are excluded from the model iterations by giving them a prior outlier probability of 100% because of the poor collagen preservation. The chronology is compared to the North Greenland Ice Core Project (NGRIP) Greenland Ice Core Chronology 2005 (GICC05) (43) δ18O paleo-environmental record with Greenland Interstadials (GI) 12 to 3 and Heinrich events (H) 5, 4, and 3 indicated.

Fig. 3 shows the plots of stratigraphically distinct lithic artifacts with associated radiocarbon dates. The levels display a high degree of lithic assemblage integrity supported by the technological characteristics, raw material representation, and systematic artifact refitting. The radiocarbon dates also reflect stratigraphic integrity with no significant inversions between the levels. Furthermore, the dated bone samples have fresh, well-preserved green bone fractures with no signs of trampling or abrasion (*SI Appendix*, Figs. S4–S6) and were taken from deposits with a low (2°–4°) inclination (40). The sedimentological and geochemical details published in Benedetti et al. (40) provide additional support for the integrity of the deposits, which are evidenced by distinct beds, varying from roughly 5 to 30 cm thick, with uniform properties of clast size and fine sediment content. Thus, vertical and nonvertical migration in the matrix appears to have been negligible. The dates reported here are included with previous determinations in a Bayesian model constructed using the new IntCal20 in the OxCal program 4.4 (41, 42) (Fig. 4 and *SI Appendix*, Table S2). The temporal gaps between the layers reflect the sampling of archaeological horizons within them, and the slow rate of sedimentation reported previously (40).

**Fig. 5.**
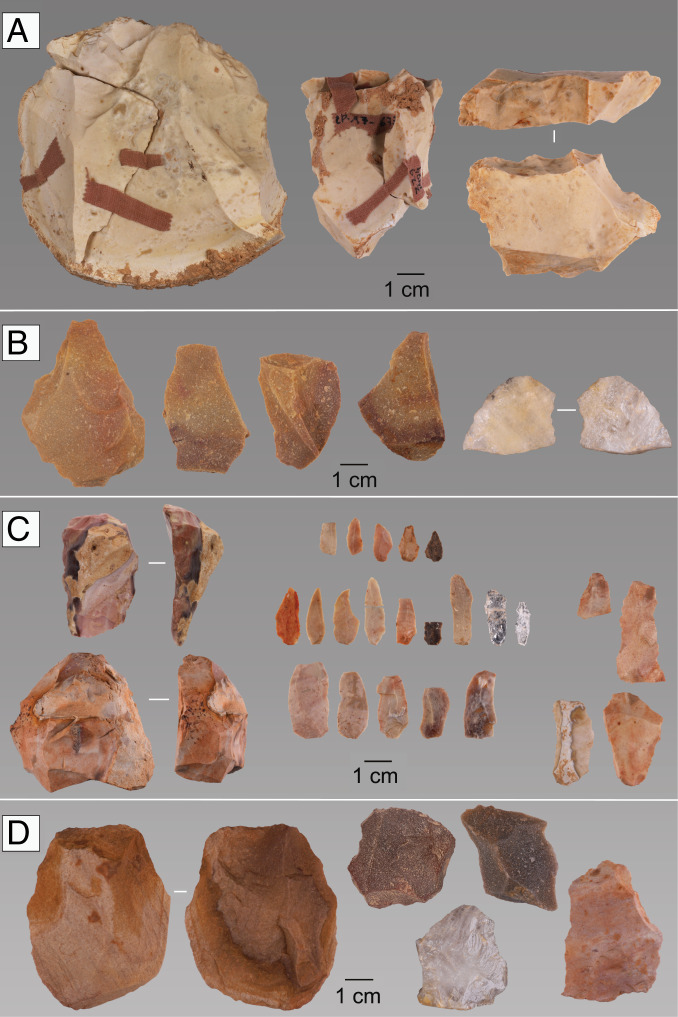
Lithic artifacts from the Middle to Upper Paleolithic transition levels at Lapa do Picareiro. (*A*) Chert cores and core tablet from level DD. (*B*) Quartzite flakes from level FF. (*C*) Early Aurignacian carinated endscrapers/cores and bladelets from level GG. (*D*) Middle Paleolithic core and flakes.

The base of the sequence discussed here, level JJ, is a ∼1-m-thick layer of small-medium limestone clasts with reddish-brown fine sediment (*SI Appendix*, Table S3). The deposit contains lithic artifacts made using discoidal core/flake technology typical of the Middle Paleolithic, dispersed charcoal, and animal bones. The radiocarbon samples were taken from two artifact horizons separated by a ∼20-cm-thick dark sediment lens. The lower horizon dates to ∼51.5 to 44.1 ka cal BP and the upper horizon dates to ∼45.0 to 43.5 ka cal BP. The top 20 to 30 cm of level JJ, dated 42.9 to 42.4 ka cal BP, contains bones with percussion marks consistent with intentional butchery by humans but lithic artifacts have not yet been found in this zone (Fig. 5*D*).

Levels II through GG represent a ∼40-cm-thick, distinct, and partially cemented portion of the sequence that caps level JJ. The lower part, level II is a layer of medium and larger clasts with brown mud cemented by calcite followed by a layer of medium-sized clasts with brown mud that is uncemented, level HH. Level GG is a mostly cemented layer of larger limestone clasts and brown fine sediment that extends across the center of the cave. In the back of the cave, the calcite cement gradually disappears. The thick, firmly cemented parts of level GG in the center of the cave appear to have formed by postdepositional precipitation from calcium-enriched water penetrating the interstitial spaces between limestone clasts on the cave floor (*SI Appendix*, Fig. S4). A sharp peak in magnetic susceptibility values recorded in level II indicates a brief warm period followed by low values in levels HH and GG, corresponding to a period of prolonged cold, dry conditions during its formation (40). The precipitation of carbonate cement likely occurred at the end of this cold episode with the return of humid conditions. The earliest Aurignacian artifacts are distributed throughout the muddy matrix from the base of the large clasts of level GG through level II (Fig. 2 *C*–*E* and *SI Appendix*, Fig. S7). The radiocarbon samples were taken from the uncemented areas of level GG and II, in direct association with the levels GG-II artifacts.

Levels GG-II contain a diagnostically Upper Paleolithic lithic assemblage comprised of small bladelets and carinated endscrapers (now recognized as cores) typical of the early Aurignacian (6, 44, 45) (Fig. 5*C*). The bladelets have the characteristic dimensions and shape, lacking retouch, similar to those found in the early Aurignacian assemblages of northern Iberia (46). This assemblage is made primarily from chert but also includes a small number of quartz flakes. Accelerator mass spectrometry (AMS) radiocarbon dating of anthropically modified, medium-sized ungulate bones provide bracketing ages of ∼41.9 to 41.1 ka cal BP (*terminus* *post quem* or earliest possible date) and 39.4 to 38.1 ka cal BP (*terminus ante quem *or latest possible date). Thus, the assemblage falls within most of the Proto-Aurignacian and entirely within the Early-Aurignacian time frame in Europe (11, 47, 48). The artifact deposition likely took place during the Greenland Stadial (GS)-9 climate phase associated with Heinrich Event 4 (H4) (39.9 to 38.2 ka) or GS-10 (40.8 to 40.1 ka), but possibly even earlier during the time of Greenland Interstadial (GI)-10 (41.4 to 40.8 ka) and GS-11 (42.2 to 41.5 ka) (43). Most of the artifacts plot within a ∼20-cm linear band between the dated samples, making a more exact age estimation or paleoclimatic association difficult at this time, but these still represent the oldest, most precise, and reliable dates for the Aurignacian in western Iberia.

In stark contrast, overlying sediments of level FF are composed of loose, small-medium limestone clasts and dark reddish-brown fine sediment. This layer contains undiagnostic quartz and quartzite flakes, animal bones, and dispersed charcoal dated 38.6 to 36.4 ka cal BP (Fig. 5*B*). The organic matter content and high magnetic susceptibility values in level FF reflect a period of relatively mild climate associated with GI-8 (43).

Level EE is a thin layer (5 to 10 cm) of small clasts with reddish-brown fine sediment that appears to be archaeologically sterile. The radiocarbon date for level EE (36.7 to 36.1 ka cal BP) is indistinguishable from those of level FF. The dated bone sample plots in line with those from level FF and, therefore, the MAMS-44444 (Mannheim AMS lab at the Curt-Engelhorn-Centre for Archaeometry) specimen may not accurately date level EE.

Level DD is a 20- to 30-cm-thick layer of medium-large limestone clasts with brown fine sediment. This layer, dated 35.4 to 34.8 ka cal BP, contains a lithic assemblage almost exclusively on chert, characterized by the production of large flakes using prismatic core technology (Fig. 5*A*). The dates for these levels place them chronologically within the Aurignacian time frame in western Europe (47, 48) but the assemblages lack diagnostic pieces for a particular phase. Low magnetic susceptibility values and radiocarbon dates correspond to GS-8 (43).

Despite their small size, comparison of the lithic assemblages reveals important differences in the frequencies of the different classes of blanks across all levels (*SI Appendix*, Table S4). While levels JJ, FF, and DD are dominated by complete flakes and flake fragments, the level GG-II assemblage is marked by a high frequency of bladelets and bladelet fragments. Blades are also present in the level GG-II assemblage but completely absent from all of the remaining levels assessed here. Overall, cores and retouched tools are very rare in all assemblages (*SI Appendix*, Table S5). The levels also differ in lithic reduction strategies. In level JJ, centripetal, subcentripetal, and other reduction patterns are equally represented, typical of a Mousterian assemblage, while for all of the overlaying levels unidirectional strategies are predominant.

Raw material use is also markedly different across levels (*SI Appendix*, Fig. S8). Levels JJ and FF are characterized by the use of quartzite and quartz, with chert representing only 15% in the Mousterian level JJ, and completely absent from level FF. In levels GG-II and DD, the scenario is totally different as chert is the most frequent raw material (>75% of all artifacts), with milky quartz/rock crystal (in GG-II) and quartzite (in DD) making up the rest of the assemblages. A noteworthy number (*n* = 12) of chert artifacts in level DD refit into four different sets, revealing both the integrity of the assemblage and the occurrence of onsite knapping activities. While no refits have been made among the chert bladelets and the carinated endscrapers recovered in level GG-II, comparison between the bladelets and the last scar on the flaking surface of the carinated elements suggest a single reduction sequence for producing small bladelets (*SI Appendix*, Table S6).

Abundant faunal remains have been recovered in all of the levels presented here. Preliminary results of ongoing analyses indicate that large and small mammal taxonomic representation changed very little across the Late Middle and Early Upper Paleolithic layers. *SI Appendix*, Tables S7–S10 provide results of taxonomic identifications made to date. Large and small mammals for all levels include red deer, ibex, and rabbit. Some of the ungulate remains show evidence for butchery with cut marks, percussion scars, and long bone fractures consistent with marrow removal. Horse was also exploited in the Late Middle Paleolithic level JJ. The rabbits show little if any direct evidence for human exploitation but only a very small proportion of the assemblage has been analyzed in detail. Carnivores are primarily represented by lynx in levels DD-FF and JJ. Fox also appears in the Late Middle Paleolithic. A variety of bird taxa are also present in the assemblages, but it is not clear if the remains were brought to the cave by humans. Ongoing taphonomic analyses should resolve this issue. The same can be said for the micromammal and herpetological taxa.

## Discussion

The stratigraphy, techno-typological analysis of the lithic artifacts, and radiocarbon dating demonstrate that the level GG-II assemblage represents a discrete occupation layer, wholly distinct from those in the levels above and below. The assemblage is small but consistent with the attributes of the early Aurignacian. The dates presented here conservatively place the level GG-II occupation at ∼41.1 to 38.1 ka cal BP.

Our results from Picareiro provide definitive evidence that modern humans were in western Iberia at a time when, if present at all, Neanderthal populations would have been extremely sparse. Our data offer some resolution to the implications of Wood et al. (29) scenarios for the ∼42 to 37 ka cal BP time frame. We can discount their scenario in which southern Iberia was abandoned by both Neanderthals and modern humans and confirm the one in which modern humans spread into the southern regions soon after they arrived in northern Iberia. As for the scenario in which Neanderthals were present in southern Iberia until very late, the Picareiro data alone cannot resolve this issue. Middle Paleolithic occupations at Picareiro ended by 42.5 ka cal BP, but they apparently continued until ∼36 ka cal BP at Gruta da Oliveira just 4.2 km away, based on current evidence (49, 50).

Our results also cast further doubt on the idea of a “hard border” or frontier between Neanderthal and modern human populations between ∼42 and 37 ka cal BP (22, 51). Instead, the Ebro river valley was likely a permeable landscape feature that facilitated dispersal (52). Modern humans may have encountered a few remnant Neanderthal groups but it appears that most of Iberia south of the Ebro was already depopulated (35). This pattern is evident in Portugal, where Middle Paleolithic end dates cluster at ∼45 to 42 ka at Foz do Enxarrique (30), Mira Nascente (53), Lapa do Picareiro, and Cardina (54), followed by a nearly total absence of evidence for Neanderthals on the landscape.

The Picareiro data confirm a rapid modern human dispersal across Iberia that opens up additional lines of inquiry, testable hypotheses, and explanatory scenarios for the Middle to Upper Paleolithic transition. First, the early Aurignacian arrival indicates a substantial time gap between the Picareiro record of Early Upper Paleolithic occupations and the rest of the region (27, 54). This may reflect an expansion of small pioneer groups that did not leave a highly visible footprint due to low population density (55) or did not establish a permanent foothold in the region. Alternatively, climate-induced erosive episodes may have erased much of the archaeological evidence of their presence on the landscape (37, 38). Either case limits the detectability of the earliest pioneers. Another possibility is that the evidence exists among assemblages in the region lacking diagnostic elements and/or radiometric dates. Picareiro level FF, dated within the Evolved Aurignacian time frame, exemplifies this case with simple flakes, mainly quartzite and quartz, and no formal tools. Aside from its stratigraphic position and radiocarbon dating, there is nothing diagnostically Aurignacian about the assemblage. Level FF represents a low-cost, expedient technology often attributed to the Middle Paleolithic in Iberian sites dated ∼42 to 32 ka cal BP (56). Assemblages like these may have been a regular element of the pioneer phase in modern human dispersal (55). Just how widespread and common they were during the Aurignacian time frame remains to be investigated but similar expedient core reduction strategies are known throughout the Upper Paleolithic at many sites (57), including Picareiro.

Second, the successive climatic downturns between 44 and 40 ka may have created new opportunities for modern human dispersal into the region, as predicted by Banks et al. (47), possibly following the southward range expansion of familiar Eurosiberian taxa along the Atlantic margin or the major east-west river drainages such as the Duero or Tagus (*SI Appendix*, Fig. S9). River systems like these played a key role as “communication corridors and mobility conduits” for the dispersal of modern humans across Europe (52). A critical aspect of dispersal would have been the development of cognitive maps to navigate unknown landscapes, and rivers are the easiest spatial features to follow (58). New optically-stimulated luminescence (OSL) ages for Cardina, an open-air site in the Douro drainage basin of northeast Portugal, show a long hiatus between the last Middle Paleolithic occupation ∼42.9 ka and the Evolved Aurignacian occupation ∼33.6 ka (54). The absence of an earlier Aurignacian occupation could rule out the Douro valley as a dispersal route, but the area may eventually yield supportive evidence. Rather, the spread of modern humans across the Iberian Peninsula may represent a “jump dispersal” through which they avoided or bypassed unproductive or high-risk areas like the more arid interior of Iberia (2). Along interior river drainages, rapid dispersal may have been necessary to mitigate water scarcity during extreme droughts associated with Heinrich events, thus explaining the lack of early Aurignacian sites. Alternatively, the coastal route hypothesis advanced by Cortés-Sánchez et al. (23) is also supported by the distribution of Early Upper Paleolithic sites along the Iberian coast (59). This ecotonal position likely provided more predictable resources and less ecological risk during periods of climatic stress. The topography of coasts would have also facilitated communication and transmission of information among pioneer groups (55).

Third, Neanderthals and modern humans may have been contemporary and in close proximity in the limestone massif of Estremadura in Portugal. If so, there is no evidence that they were in direct contact as the Picareiro GG-II occupation took place between the occupations of Gruta da Oliveira levels 9 and 8. However, level FF, without diagnostic artifacts, is contemporary with Oliveira level 8 and could indicate either coexistence or successive, alternating presence of different populations. The presence of modern humans overlapping in time lends support to competitive exclusion as an explanation for Neanderthal extinction (60, 61). On the other hand, if the Gruta da Oliveira level 8 dates are erroneously too young, as postulated by Wood et al. (29), then there are no Neanderthal or Middle Paleolithic sites in Portugal that postdate ∼42 ka cal BP. Thus, there may be no temporal overlap or competition between the last Neanderthals and earliest modern humans in the region.

Lastly, the Picareiro record appears to reflect the pattern across much of western Eurasia where sterile layers between the last Neanderthal and modern human occupations have been linked to millennial-scale climate cycles and environmental change (62). Depopulation appears to have occurred during severe cold and dry stadials that disrupted and fragmented habitat patches across western Eurasia, negatively impacting Neanderthal populations and opening new spaces for modern human dispersal (63). In Iberia, paleoclimate records show regional variability in the terrestrial response to GS-12 (44.3 to 43.3 ka), GS-11 (42.2 to 41.5 ka), and GS-9 or HS-4 (39.9 to 38.2 ka) (64, 65). The archaeological and sedimentological data from Picareiro and other sites are too coarse grained at present, but the timing of these successive perturbations appear to coincide with regional Neanderthal depopulation. At Picareiro, the last Middle Paleolithic occupation corresponds to the beginning of GS-12, followed by an apparent occupational hiatus in the upper 20 to 30 cm of level JJ, with the subsequent Aurignacian arrival between GS-11 and GS-9.

Although we may never know the first actual presence (2) of modern humans or the last actual presence of Neanderthals in Iberia, south of the Ebro basin, the data from Lapa do Picareiro expand our knowledge about the dispersal of modern humans. Based on current understanding of Middle and Upper Paleolithic technological associations, the Picareiro case provides definitive evidence for the early appearance of modern humans in westernmost Eurasia, disrupting previous models and creating opportunities for new lines of inquiry. A major gap in our knowledge of the 42 to 37 ka cal BP interval remains to be filled with further investigation and continued field work.

## Materials and Methods

### Excavation Methodology.

The excavation is laid out using a 1 × 1 m grid system. Each unit is excavated according to the natural stratigraphy and 5-cm artificial levels are used within the thicker levels. Artifacts, bones, features, and stratigraphic topography are mapped in three dimensions using a total station. All sediment is sieved through 2- and 4-mm mesh screens. The excavators embed screens to separate the finer sediment from the larger fraction which is mostly limestone éboulis. The larger fraction is sorted in the field. The remaining sediment in the 2-mm screen is water-sieved in the laboratory in order to recover small bones, lithics, and macrobotanical remains. This allows for the recovery of micromammal, amphibian, and bird bones, shell fragments, stone chippage, personal ornaments, and charcoal.

### Stratigraphy.

Stratigraphic level designations were based on changes in clast size, color, mud content, and firmness (40). Sediment samples were collected at 10-cm-depth intervals from the top of the sequence. Clasts were assigned a modal clast size category (very small to very large) and samples were measured to calculate a mean large clast size (in millimeters). Analyses were completed on the <2-mm fraction of the profile samples at the University of North Carolina Wilmington Soils and Sedimentology Laboratory.

### Artifact Analysis.

Lithic assemblages were analyzed using a techno-typological approach focused on raw material use, refitting, core preparation, and blank or flake attributes including platform type, dimensional analysis, and retouched tools typology. Artifact analyses were done at the Interdisciplinary Center for Archaeology and Evolution of Human Behavior (ICArEHB) at the Universidade do Algarve.

### Faunal Analysis.

Taxonomic identification of the faunal remains was done using comparative reference collections at the Laboratório de Arqueociências of the Direcção-Geral do Património Cultural in Lisbon and the Estación Biológica Doñana of the Consejo Superior de Investigaciones Científicas in Seville. This work established the taxonomic composition of the assemblages based on limited analyses of the piece-plotted remains. Only preliminary results of ongoing analyses are presented here.

### Radiocarbon Pretreatment.

Bone samples were pretreated at the Department of Human Evolution at the Max Planck Institute for Evolutionary Anthropology (MPI-EVA), Leipzig, Germany, using methods described previously (66). First, the outer surface of the bone samples were cleaned by a shot blaster, and then 500 mg of bone was taken. The samples were decalcified in 0.5 M HCl at room temperature until CO_2_ effervescence was no longer observed, usually about 4 h. Humics were removed by adding 0.1 M NaOH for 30 min. This was followed by adding 0.5 M HCl for 15 min. The resulting solid was gelatinized at pH 3 in a heater block at 75 °C for 20 h. The gelatin was filtered in an Eeze-Filter (Elkay Laboratory Products) to remove small (**<**80 **μ**m) particles. The gelatin was then ultrafiltered using Sartorius “VivaspinTurbo” 30-kDa ultrafilters. The filter was first cleaned to remove carbon-containing humectants. The samples were lyophilized for 48 h. All dates were corrected for a residual preparation background estimated from pretreated ^14^C-free bone samples, kindly provided by the Mannheim Laboratory, Mannhein, Germany and pretreated in the same way as the archaeological samples (67). To assess the preservation of the collagen yield, C:N ratios, together with isotopic values, were evaluated. The C:N ratio should be between 2.9 and 3.6, and the collagen yield not less than 1% of the weight (68). The stable isotopic analysis was carried out at the MPI-EVA (lab code R-EVA) using a ThermoFinnigan Flash EA coupled to a Delta V isotope ratio mass spectrometer.

### Calibration and Bayesian Modeling.

We constructed a Bayesian model for the Lapa do Picareiro using all of the radiocarbon dates produced over the last 25 years of investigation at the cave. The calibration was made using the new IntCal20 curve within the OxCal 4.4 program (41, 42). We used a general t-type outlier model with a 5% prior probability for all dates, except for the four samples that did not pass the acceptable range of the evaluation criteria (yield of collagen less than 1%). In this model, boundaries were set according to the stratigraphic levels. The phases correspond to the artifact horizons plotted in the sequence.

## Supplementary Material

Supplementary File

## Data Availability

All study data are included in the article and *SI Appendix*. Additional data are available at Open Science Framework, https://osf.io/8zrqy/ (69).
