# Supplementary material for: The early Aurignacian dispersal of modern humans into westernmost Eurasia
Source: Proc Natl Acad Sci U S A. 2020 Sep 28;117(41):25414–22. doi: 10.1073/pnas.2016062117 (PMC7568277; doi:10.1073/pnas.2016062117)
Supplement: Supplementary File [file pnas.2016062117.sapp.pdf]

SI Appendix

Supplemental Figures

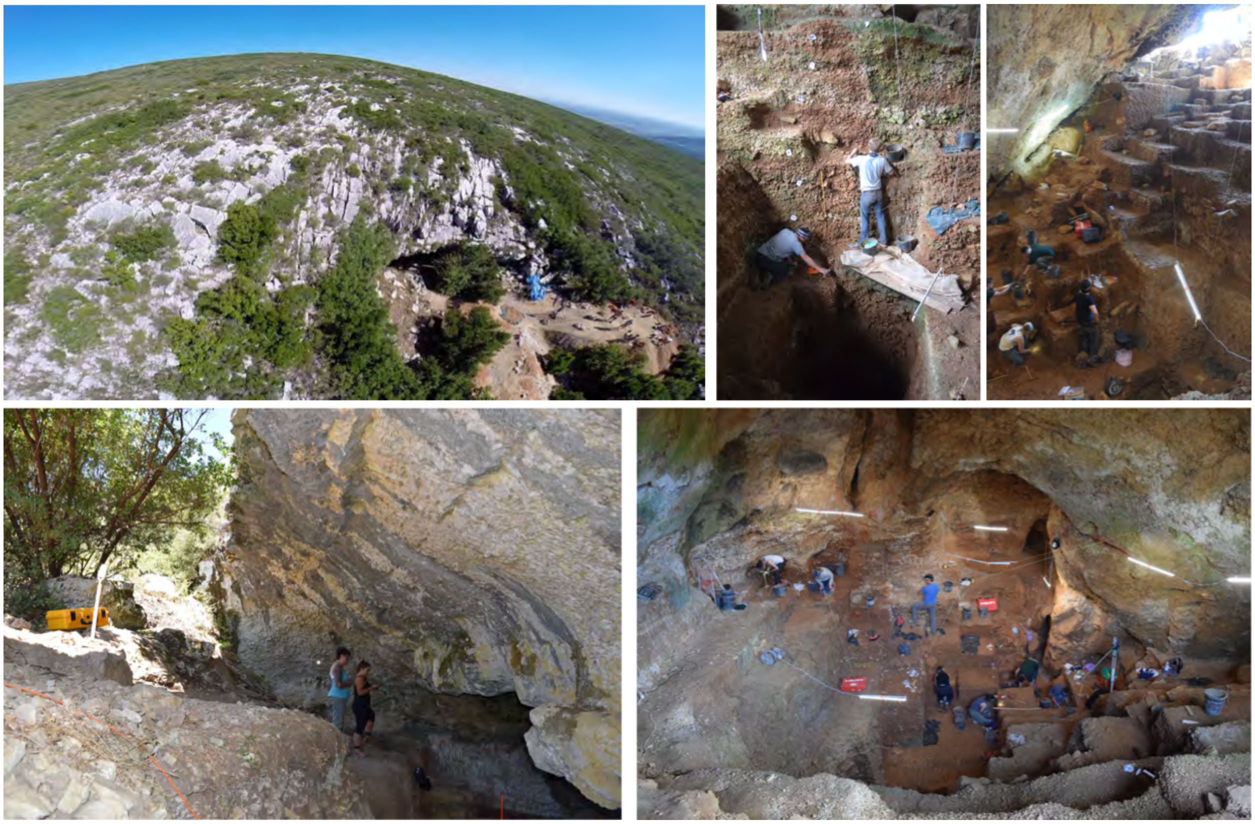

Supplementary Fig. 1: General views of cave and the excavation.

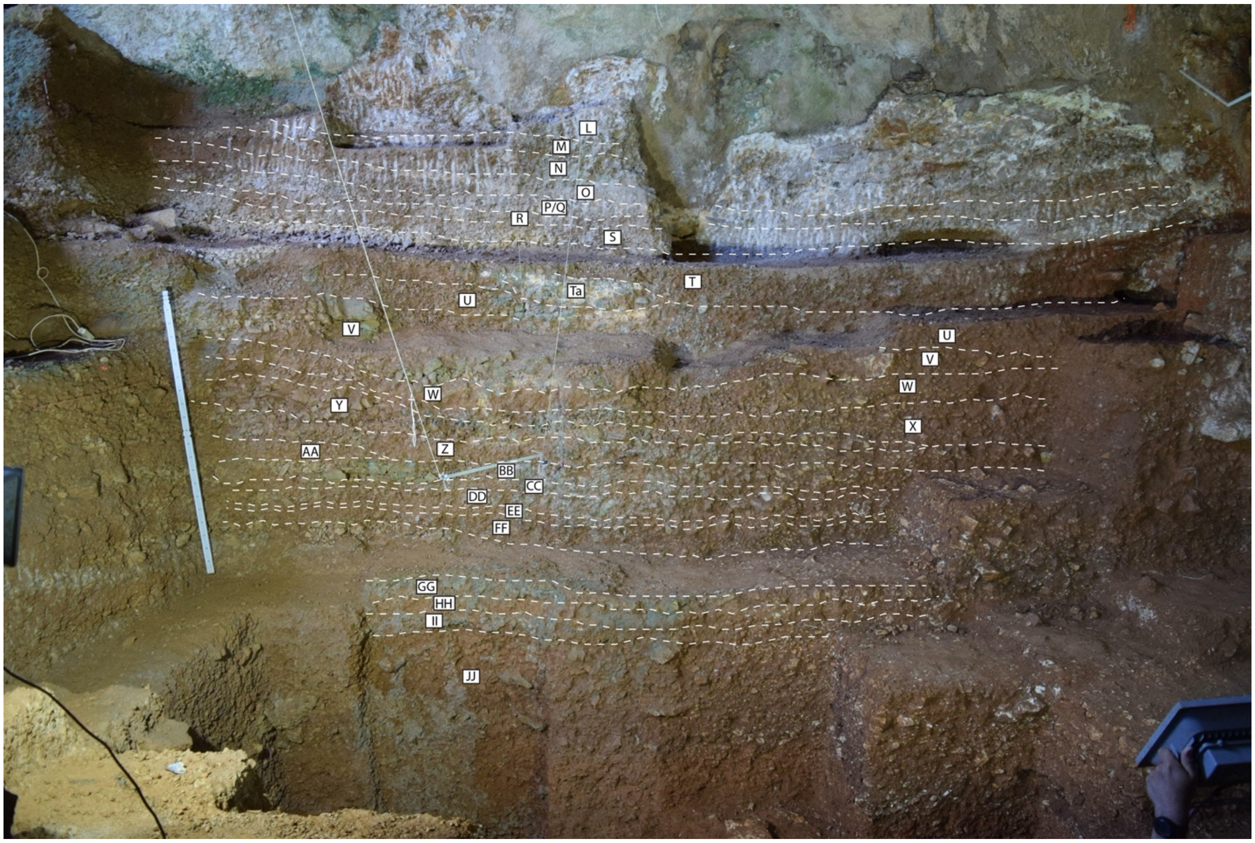

Supplementary Fig. 2: North wall profile showing Levels L through JJ. Profile corresponds to Rows 3, 4, 5, and 6 on the Y-axis and Row A, B, C, D, E, F, G, and H on the X-axis on the Figure 2 site plan.

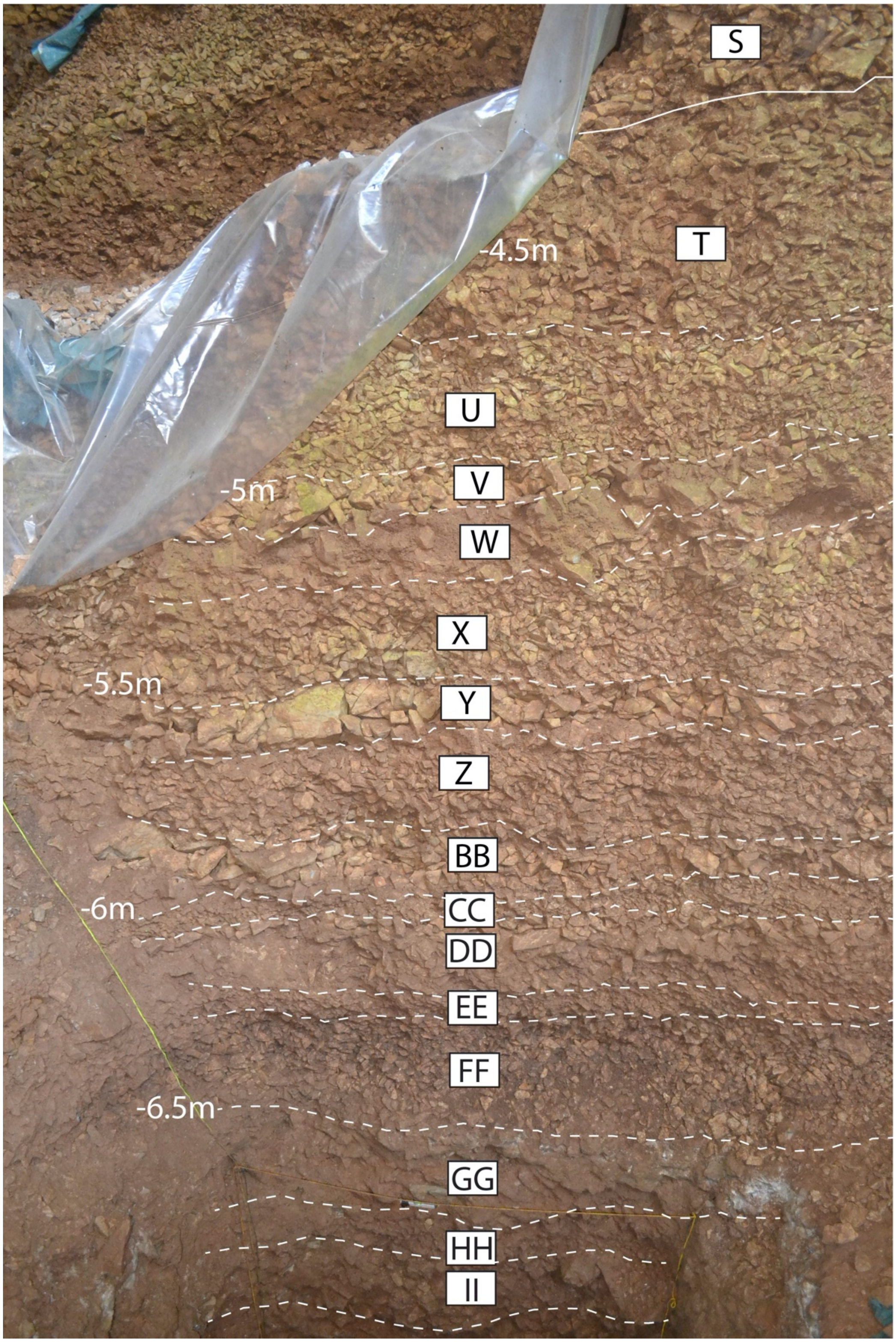

Supplementary Figure 3: Profile photo showing Levels S through JJ on the F7-E7 unit boundary.

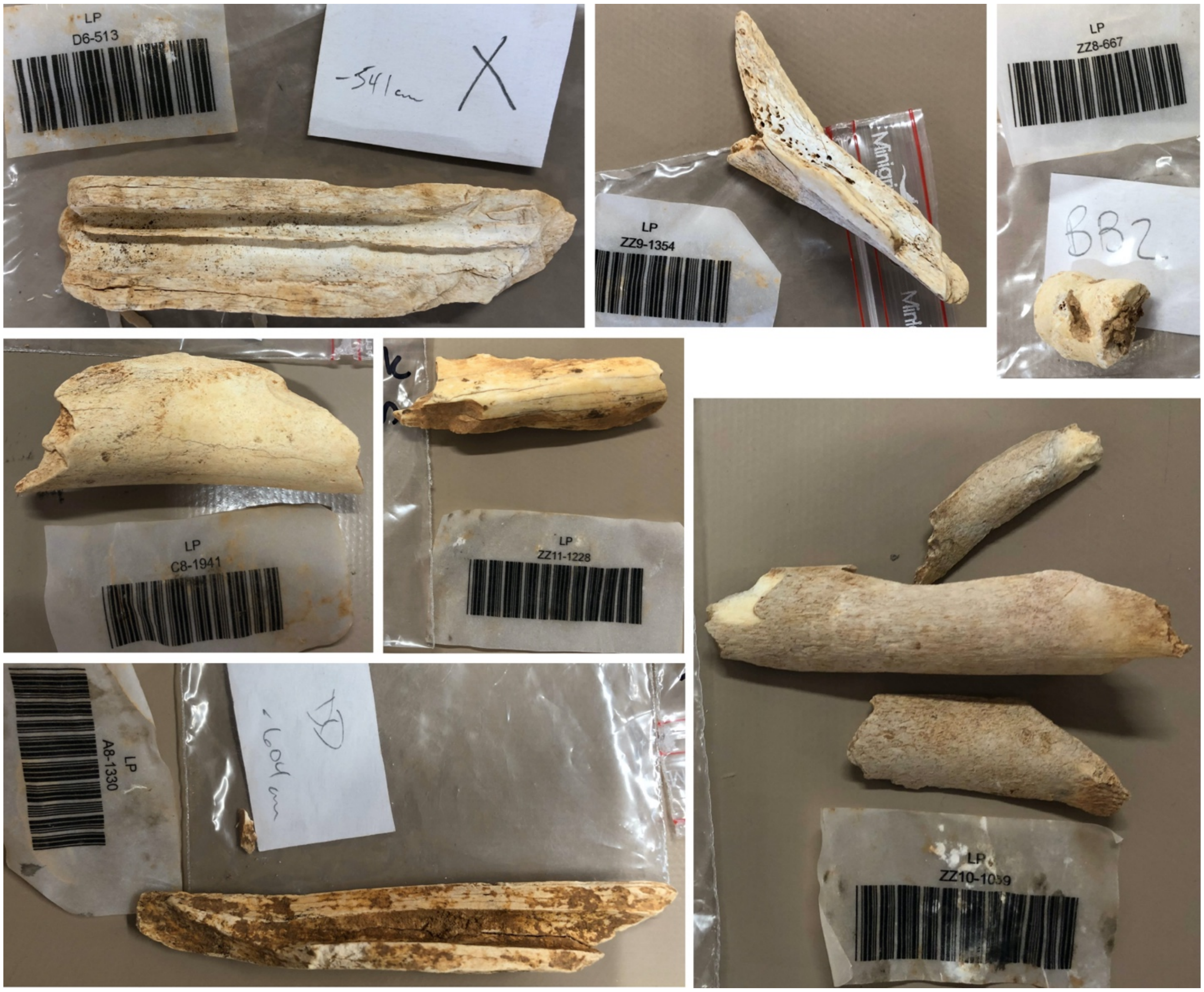

Supplementary Fig. 4. Radiocarbon samples for Levels X through FF.

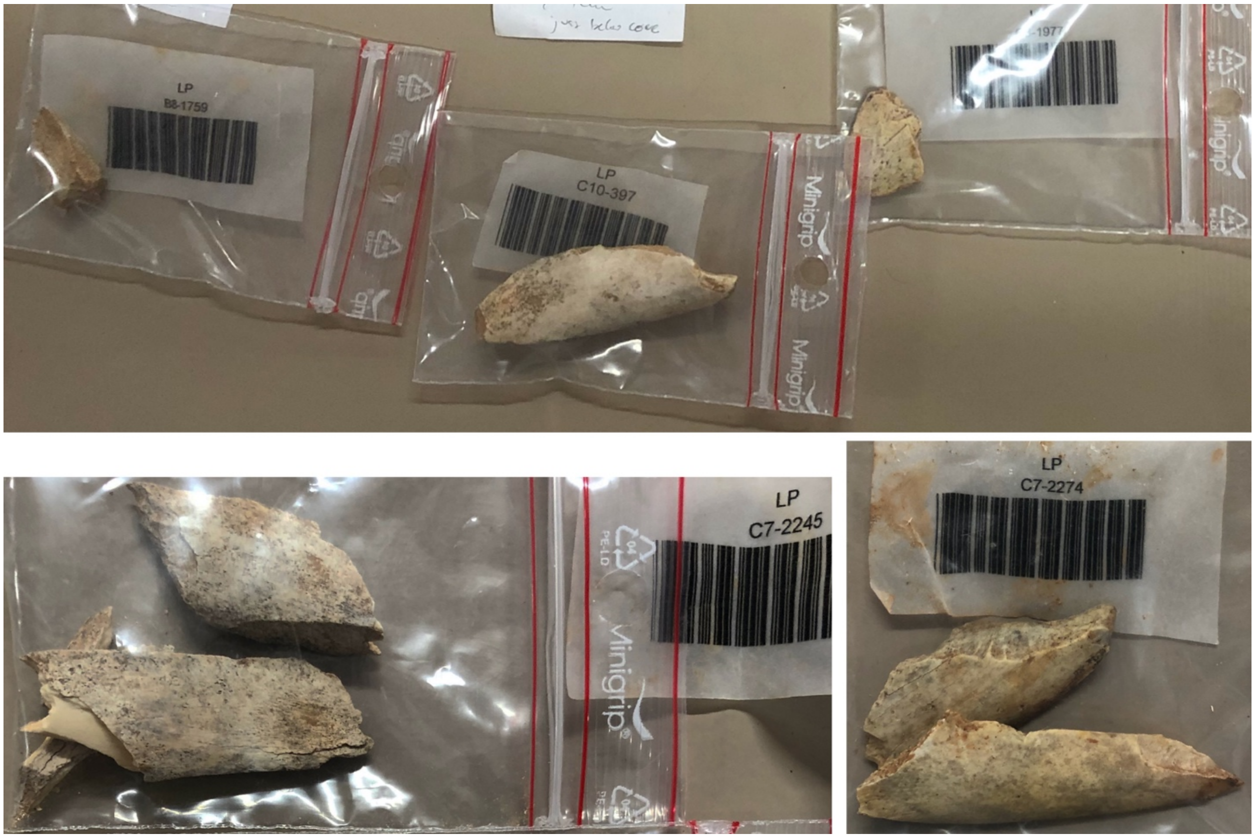

Supplementary Fig. 5. Radiocarbon samples for Levels GG-II.

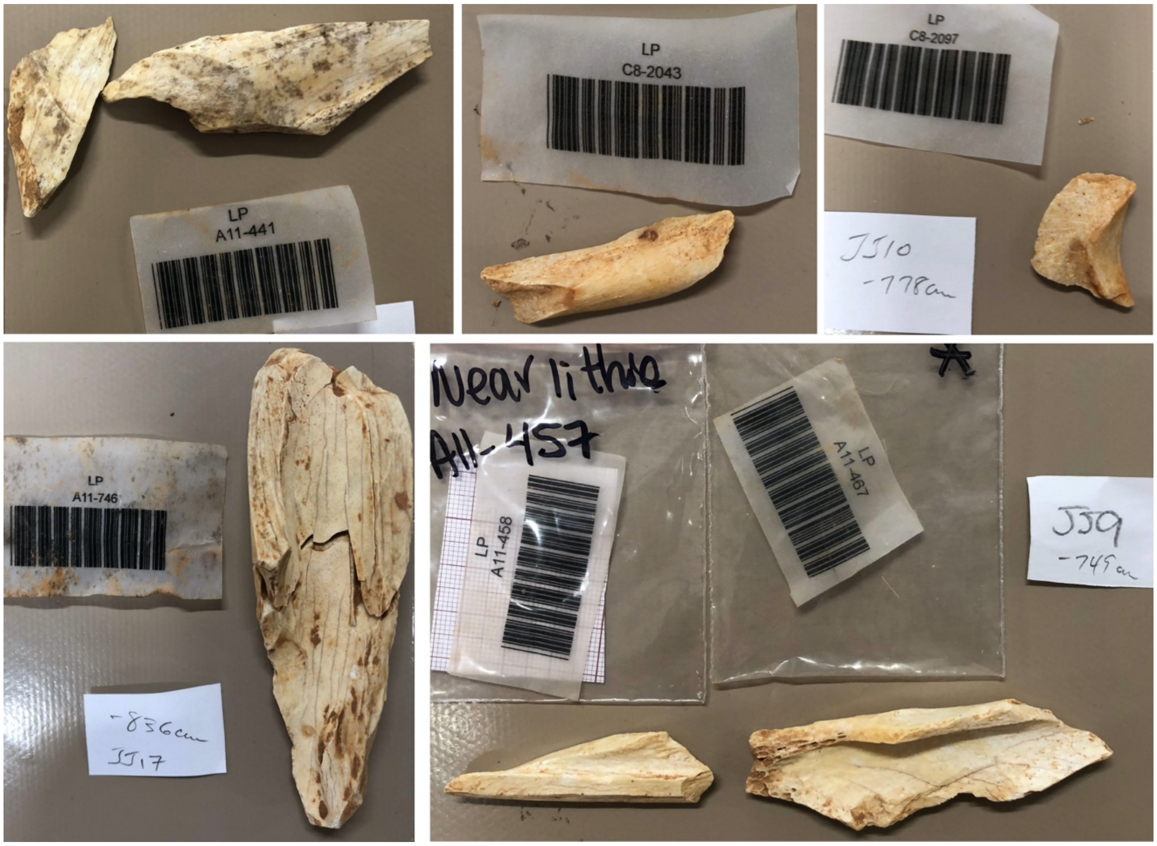

Supplementary Fig. 6. Radiocarbon samples for Level JJ.

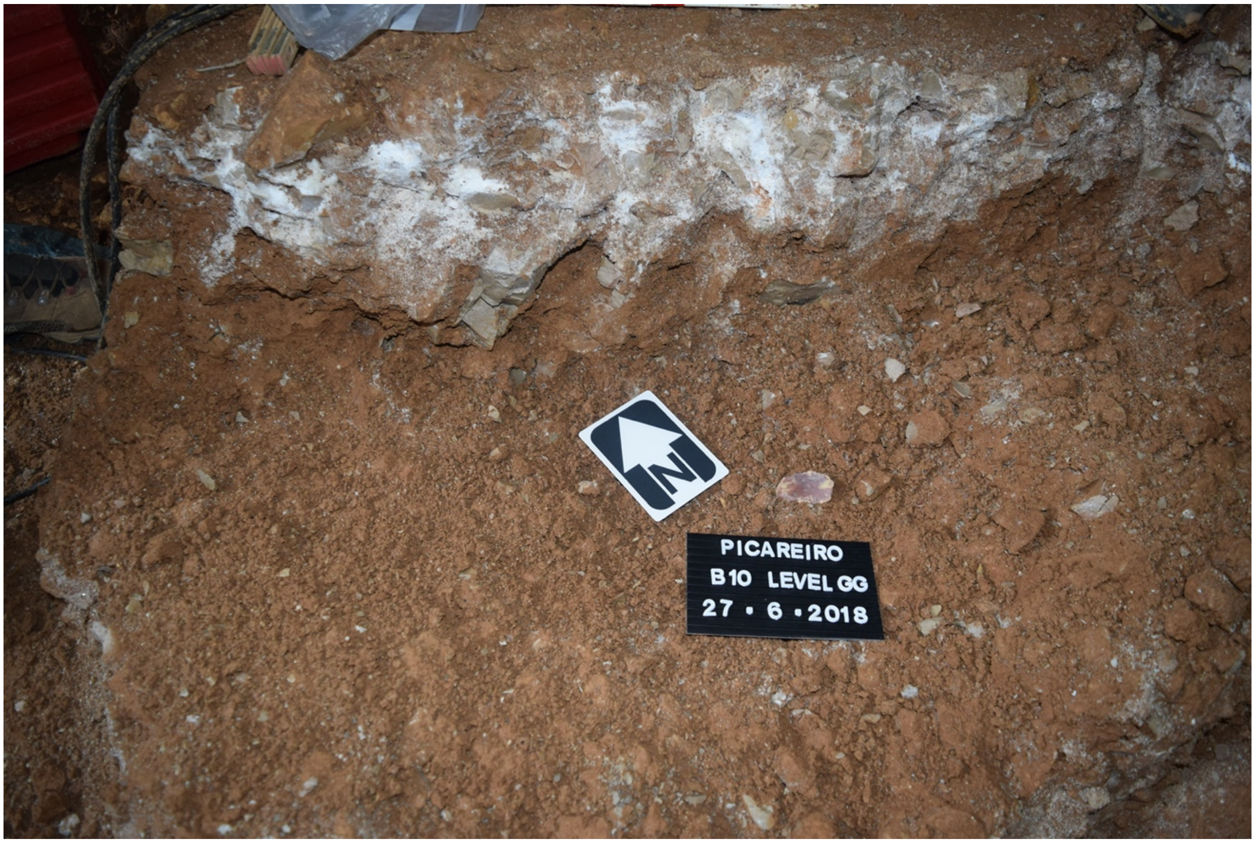

Supplementary Fig. 7. Cemented limestone clasts of Level GG in profile with exposed muddy sediments below (Level HH) containing an Aurignacian carinated endscraper/core.

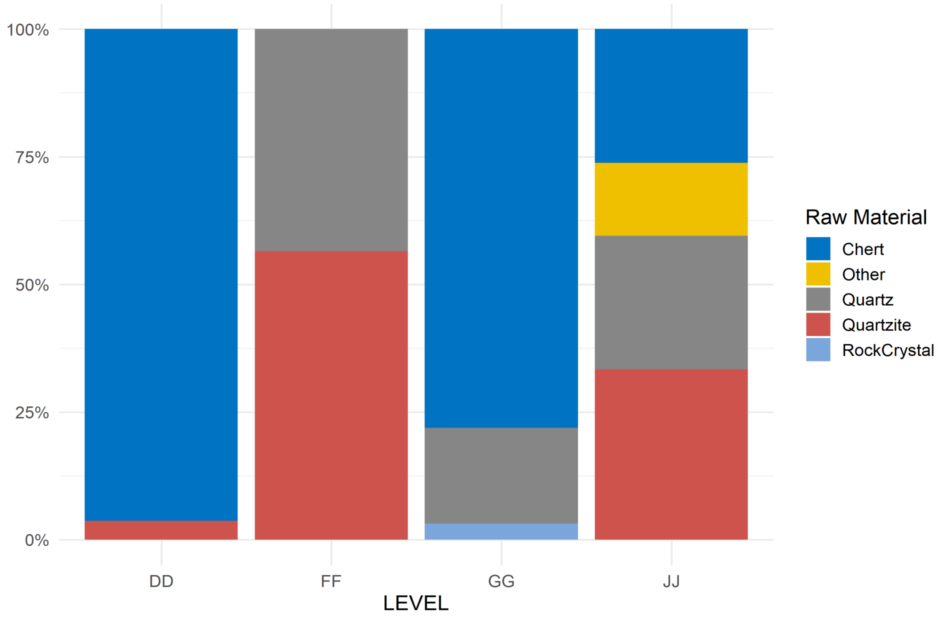

Supplementary Fig. 8. Frequencies of raw material by Level.

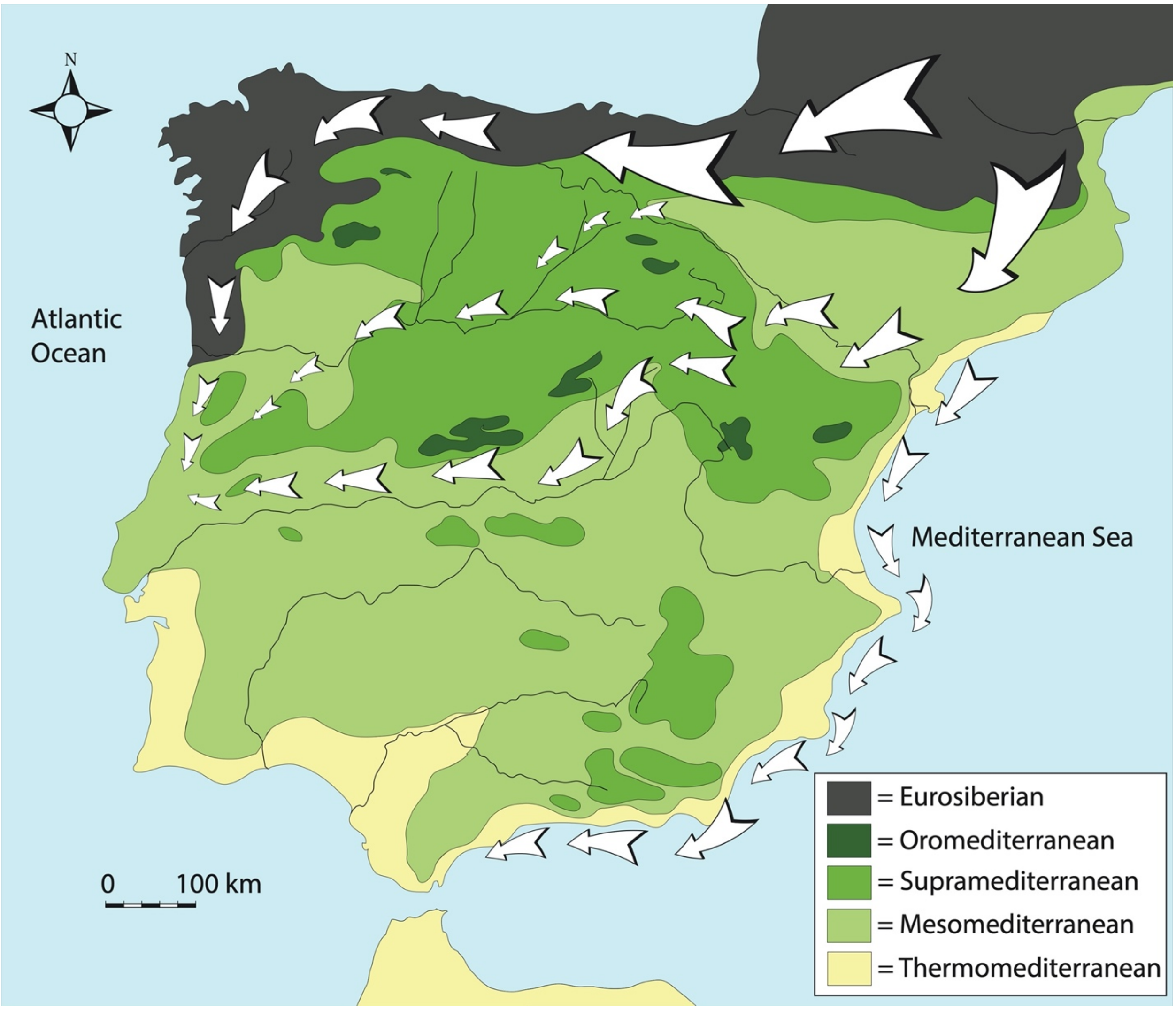

Supplementary Fig. 9: Map of Iberia showing the distribution of present-day bioclimatic zones. Arrows indicate potential modern human dispersal routes into western Iberia.

Supplementary Tables

Supplementary Table 1. Radiocarbon dates for the Middle-Upper Paleolithic transition levels in Lapa do Picareiro

MPI Code	Level	Sample ID	Archeological Attribution	Sample material	mg of collagen	% of collagen	$d^{13}C$	$d^{15}N$	%C	%N	C:N	AMS Code	^{14}C Age	1s Err
R-EVA 2977	X	D6.513	Gravettian	bone	43	7.1	-19.8	2.9	44.2	16.9	3.1	MAMS-42271	26680	80
R-EVA 2978	Z	ZZ9.1354	sterile	bone	33.3	6.0	-18.9	4.9	42.5	16.3	3.0	MAMS-42272	28330	90
R-EVA 2979	BB	ZZ8.667	nondiagnostic	bone	46.6	8.6	-19.4	2.5	44.6	17.0	3.1	MAMS-42273	30560	120
	BB		nondiagnostic	bone		0.2	-19.4		42.93		3.24	Wk-32281	30027	207
R-EVA 2981	CC	C8.1941	sterile	bone	15.6	2.7	-19.4	3.0	38.6	14.9	3.0	MAMS-42274	30260	130
R-EVA 2982	DD	A8.1330	Aurignacian	bone	8.2	1.5	-20.4	4.0	37.7	14.3	3.1	MAMS-42275	30790	120
R-EVA 2983	EE	ZZ10.1228	sterile	bone	33.2	5.2	-19.9	2.9	44.1	16.6	3.1	MAMS-44444	32400	140
	FF		nondiagnostic	charcoal			-24.0		57.04			UGAMS-20479	32200	90
R-EVA 2984	FF	ZZ10.1059	nondiagnostic	bone	30.9	4.7	-20.8	3.3	42.8	16.2	3.1	MAMS-42276	32340	140
	FF		nondiagnostic	charcoal			n/a					Wk-41259	33238	419
	FF		nondiagnostic	bone		0.27	-19.3		43.02		3.25	Wk-32219	32997	263
	FF-GG-contact		nondiagnostic	bone		0.03	-19.0		38.74		3.44	Wk-41258	32063	336
R-EVA 2988	GG-II (GG)	B8.1759	early Aurignacian	bone	39	6.6	-20.5	6.1	43.9	16.5	3.1	MAMS-42277	33910	160
R-EVA 2993	GG-II (GG)	C8.1977	early Aurignacian	bone	20.9	4.0	-19.6	3.8	39.3	14.9	3.1	MAMS-42281	33790	190
R-EVA 2990	GG-II (GG)	C7.2245	early Aurignacian	bone	8	1.5	-19.7	4.7	40.0	14.1	3.3	MAMS-44445	33880	160
	GG-II (HH)		early Aurignacian											
R-EVA 2994	GG-II (II)	C10.397	early Aurignacian	bone	15.4	2.8	-20.0	4.2	36.7	14.0	3.1	MAMS-42282	36670	220
R-EVA 2991	GG-II (II)	C7.2274	early Aurignacian	bone	33.5	6.1	-19.5	1.9	44.2	16.5	3.1	MAMS-42278	36390	210
R-EVA 2999	JJ	C8.2006	upper/sterile	bone	36.7	6.2	-20.0	3.0	41.1	15.6	3.1	MAMS-42283	39270	290
R-EVA 3002	JJ	C8.2043	upper/sterile	bone	44.3	7.4	-19.6	2.8	40.6	15.6	3.0	MAMS-42284	38990	280
R-EVA 3003	JJ	A11.441	Mousterian	bone	22.1	4.3	-19.9	1.9	39.1	15.0	3.0	MAMS-42285	41360	370

	JJ		Mousterian	bone		0.15	-19.9		39.53		3.4	Wk-28844	40078	1239
	JJ		Mousterian	charcoal			-25.1		55.54			UGAMS-07769	41480	220
R-EVA 3005	JJ	A11.467	Mousterian	bone	27.3	5.1	-19.9	3.6	40.0	15.4	3.0	MAMS-42286	41980	400
R-EVA 3007	JJ	C8.2097	Mousterian	bone	44.9	8.8	-19.6	3.0	39.8	15.2	3.0	MAMS-42289	47510	760
R-EVA 3009	JJ	A11.746	Mousterian	bone	20.5	3.4	-19.8	3.4	38.2	14.7	3.0	MAMS-42290	48000	820

Supplementary Table 2. Calibrated and modeled dates using IntCal20 in OxCal 4.4[40,41].

**Lapa Do Picareiro**	**Unmodelled (BP)**				**Modelled (BP)**			
Indices<br>Amodel 67.1<br>Aoverall 72.4	**Cal BP 68.2%**		**Cal BP 95.4%**		**Cal BP 68.2%**		**Cal BP 95.4%**	
	**from**	**to**	**from**	**to**	**from**	**to**	**from**	**to**
End X Gravettian					**31060**	**30210**	**31120**	**28560**
**MAMS-42271 (26680;80)**	31060	30910	31120	30830	31070	30920	31120	30830
X								
Transition Z/X Gravettian					**31950**	**30950**	**32620**	**30890**
**MAMS-42272 (28330;90)**	32850	32080	32970	31970	32880	32110	32990	31980
Z								
Transition BB/Z					**34770**	**33820**	**34880**	**32610**
**MAMS-42273 (30560;120)**	35140	34730	35280	34610	34820	34560	35030	34430
Wk-32281 (30027;207)	34640	34300	35030	34110	34730	34360	35000	34020
BB								
Transition CC/BB					**34940**	**34610**	**35130**	**34510**
**MAMS-42274 (30260;130)**	34760	34460	35110	34370	35060	34670	35180	34580
CC								
Transition DD Aurignacian/CC					**35210**	**34800**	**35330**	**34640**
**MAMS-42275 (30790;120)**	35340	34910	35440	34730	35390	35080	35480	34830
DD								
Transition EE/DD Aurignacian					**36520**	**35230**	**36620**	**35050**
**MAMS-44444 (32400;140)**	36900	36500	37050	36320	36570	36310	36730	36190
EE								
Transition FF/EE					**36670**	**36390**	**36820**	**36270**
**UGAMS-20479 (32200;90)**	36630	36320	36830	36230	36820	36530	36920	36400
MAMS-42276 (32340;140)	36840	36440	36990	36290	36900	36580	37030	36430
Wk-41259 (33238;419)	38710	37260	39260	36860	37890	36750	38600	36550
Wk-32219 (32997;263)	38050	36980	38820	36770	37650	36760	38370	36530
Wk-41258 (32063;336)	36810	36110	37180	35640	36990	36510	37630	36330
FF								
Transition GG early Aurignacian/FF					**39130**	**37520**	**39180**	**36820**
**MAMS-42277 (33910;160)**	39380	38890	39510	38410	39310	38820	39420	38420
MAMS-42281 (33790;190)	39270	38560	39410	37900	39240	38670	39380	38140
MAMS-44445 (33880;160)	39350	38820	39490	38330	39290	38800	39400	38390
GG								
Start GG early Aurignacian					**39620**	**39020**	**40160**	**38650**

**End**					**40440**	**39340**	**41090**	**39060**
**HH**								
Start HH early Aurignacian					**41280**	**40210**	**41530**	**39570**
**End**					**41600**	**41070**	**41790**	**40550**
**MAMS-42282 (36670;220)**	41810	41400	41970	41230	41730	41360	41900	41240
MAMS-42278 (36390;210)	41590	41180	41830	41050	41670	41290	41840	41150
II								
Start II early Aurignacian					**41990**	**41450**	**42410**	**41310**
**End**					**42730**	**42300**	**42830**	**41850**
**MAMS-42283 (39270;290)**	42880	42590	43040	42460	42820	42580	42950	42470
MAMS-42284 (38990;280)	42760	42490	42900	42380	42770	42530	42890	42420
JJ Upper								
Start					**43050**	**42610**	**43610**	**42510**
**Transition JJ/2**					**44180**	**43180**	**44460**	**42820**
**MAMS-42285 (41360;370)**	44640	44050	44850	43380	44630	44140	44940	43530
Wk-28844 (40078;1239)	44310	42660	45620	42210	44930	43580	46190	43000
UGAMS-07769 (41480;220)	44600	44270	44770	44040	44600	44280	44770	44060
MAMS-42286 (41980;400)	45010	44440	45430	44200	44990	44420	45420	44210
MAMS-42289 (47510;760)	51410	48920	54750	48170	50320	48520	51570	44190
MAMS-42290 (48000;820)	52230	49540	...	48660	50550	48640	51800	44190
JJ								
Start Mousterian JJ					**51570**	**44490**	**53200**	**44350**

Supplementary Table 3: Properties of stratigraphic levels relevant to the Middle-Upper Paleolithic transition in Lapa do Picareiro[39].

Level	Depth interval (m)	Matrix Color (Munsell)	Sediment Description [a,b,c]	Lithic industry [d]
X	5.25-5.32	strong brown (7.5YR5/6)	Medium clasts, little fine sediment, friable, abundant small animal bones.	Gravettian
Y	5.32-5.53	strong brown (7.5YR5/6)	Medium to large clasts, little fine sediment, common bones.	None
Z	5.53-5.71	strong brown (7.5YR5/6)	Medium clasts in muddy matrix, friable, common bones.	None
BB	5.71-5.96	strong brown (7.5YR5/6)	Large clasts, friable, few boulders up to 60 cm, large and small animal bones.	Upper Paleolithic
CC	5.96-6.03	strong brown (7.5YR5/6)	Very small clasts, friable, few bones.	None
DD	6.03-6.14	strong brown (7.5YR4/6)	Medium clasts in very muddy matrix, slightly to moderately hard, large and small animal bones.	Aurignacian
EE	6.14-6.27	reddish brown (5YR4/4)	Small clasts in muddy matrix, friable to slightly hard, few bones.	None
FF	6.27-6.45	Dark reddish brown (5YR3/4)	Medium clasts, slightly to moderately hard, abundant charcoal, and large and small animal bones	Non-diagnostic
GG	6.45-6.62	strong brown (7.5YR5/6)	Large clasts, extremely hard, cemented by calcite crystals filling void spaces, animal bones.	Aurignacian
HH	6.62-6.76	strong brown (7.5YR4/6)	Medium clasts in muddy matrix, slightly hard, large and small animal bones.	Aurignacian
II	6.76-6.87	strong brown (7.5YR5/6)	Medium to large clasts in muddy matrix, very hard, calcite cement filling void spaces, large and small animal bones.	Aurignacian
JJ	6.87-7.73	dark reddish brown (5YR3/4)	Medium to large clasts in muddy matrix, slightly hard, up to 20 cm thick lens of dark reddish-brown fine sediment with dispersed charcoal, large and small animal bones.	Mousterian
JJ	7.73-8.35	dark reddish brown (5YR3/4)	Medium clasts in very muddy matrix, slightly hard, lenses of dark reddish-brown fine sediment with dispersed charcoal, large and small animal bones.	Mousterian
KK	8.35-8.64	yellowish red (5YR5/8)	Medium to large clasts, slightly to moderately hard, few bones.	Mousterian

[a] Clast size descriptors (modal class of long axis): v. small = less than 15 mm; small = 15-30 mm; medium = 30-50 mm; large = 50-75 mm; v. large = greater than 75 mm.

[b] Fine sediment content descriptors (sediment < 2 mm, % by volume): very little fine sediment = less than 3%; little fine sediment = less than 10%; muddy matrix = 20-30%; very muddy matrix = 30-40%.

[c] Hardness descriptors: L = loose = no resistance to excavation; FR = friable = little resistance to excavation by trowel; SH = slightly hard = moderate resistance to excavation by trowel, MH = moderately hard = moderate resistance to excavation by hand pick, VH = very hard = strong resistance to excavation by hand pick; EH = extremely hard = large pick, chisel or sledge required to excavate.

[d] Cultural attribution given for levels with diagnostic artifacts.

Supplementary Table 4. Lithic artifacts by class for each Level.

**Class**	**DD**	**FF**	**GG**	**JJ**	**Total**
Flake	16	8	2	31	57
FlakeFrag	5	8	3	5	21
Blade			1		1
BladeFrag			3		3
Bladelet	2		10		12
BladeletFrag		1	7		8
RetouchedPiece		2	4	1	7
RetouchedPieceFrag	1		2		3
Core	2	2		4	8
CoreFrag		1			1
CorePreparProd	1			1	2
Hammer		1			1
Shatter	20	6	1	10	37
Chip	4	14	9		27
Total	51	43	42	52	188

Supplementary Table 5. Summary of tool type frequencies by level.

Tool type	DD	FF	GG	JJ
Atypical carinated endscraper			1	
Atypical endscraper				1
Carinated endscraper			1	
Marginally retouched bladelet			2	
Partially retouched bladelet			1	
Retouched flake		1	1	
Retouched piece	1			
Sidescraper		1		

Supplementary Table 6. Dimensions of complete bladelets from Levels GG-II.

ID	Raw material	Butt type	Profile	Thickness (mm)	Width (mm)	Length (mm)
C8.1985	Chert	Linear	Twisted	0.93	7.45	20.22
D7.500	Chert	Plain	Twisted	1.67	9.37	20.85
D8.730	Chert	Linear	Twisted	1.29	7.22	13.88
C7.2258	Chert	Plain	Curved	4.1	11.24	23.13
C8.1986	Chert	Plain	Curved	1.82	11.43	24.7
B8.758	Chert	Plain	Curved	1.68	9.57	20.09
C9.954	Chert	Linear	Curved	1.88	7.65	13.11
D8.708	Chert	Crushed	Straight	1.02	6.55	11.1
YY11.1812	Chert	Plain	Straight	1.64	7.28	25.57
YY11.1812	RockCrystal	Crushed	Curved	1.47	5.28	15.76
A6.1000	RockCrystal	Crushed	Curved	2.21	7.8	20.45

Supplementary Table 7. Large and small mammal taxa identified in Levels BB though JJ. The unidentified bone plots are those plotted using the Total Station. Thousands of uncounted and unidentified remains are not included here.

Taxon	BB	CC	DD	EE	FF	GG-II	JJ
Mammals							
Cervus elaphus	7	2	7	3	41	4	44
Capreolus capreolus							
Cervidae	1						
Capra pyrenaica	14	2	5	3	2	1	14
Rupicapra rupicapra	1						
Capridae			1	1	2		1
Sus scrofa				1	1		1
Medium ungulate	119	4	250	46	151	27	602
Equus caballus							3
Lynx pardinus			1		2		2
Felis sylvestris							
Canis lupus							
Vulpes vulpes							2
Oryctolagus cuniculus	289	114	856	141	213	18	685
Subadult *O. cuniculus*	2	1	51	10	21		74
Lepus granatensis	1					1	
Unidentified bone plots	1,295	358	1,582	511	1,688	1,561	4,672

Supplementary Table 8. Micromammal taxa from late Middle and Early Upper Paleolithic levels at Lapa do Picareiro. Thousands of uncounted and unidentified remains are not included here.

	**BB**	**CC**	**DD**	**EE**	**FF**	**GG-II**	**JJ**
**Microfauna**							
**Rodentia**							
Apodemus sylvaticus							
Arvicola sapidus			1				
Eliomys quercinus	2						1
Microtus arvalis/ agrestis	1	1	2				
Microtus lusitanicus				1			1
Microtus sp.			3	1	1		20
**Muridae**							1
**Insectivora**							
Talpa occidentalis	1		3				3
**Talpidae**			7				2
**Chiroptera**							
Unidentifed	5	3	258	28	32		661

Supplementary Table 9. Avian taxa from late Middle and Early Upper Paleolithic levels at Lapa do Picareiro.

	BB	CC	DD	EE	FF	GG-II	JJ
**Birds**							
**Galliformes**							
Alectoris rufa			10		1		1
Perdix perdix							1
**Columbiformes**							
Columba livia	1				1		
**Charadriformes**							
Pluvialis apricaria	5						
Himantopus himantopus		1					
**Passeriformes**							
Turdus pilaris	8	2	3		1		2
Turdus philomelos				1			4
Turdidae							1
Petronia petronia			1				
Emberiza calandra		1		1			
Passeriformes		1					1
**Corvidae**							
Pyrrhocorax pyrrhocorax			9		1		
Corvus monedula			1				
Corvidae	2						
**Accipitriformes**							
Aquila adalberti							5
Gyps fulvus					10		
Total indet Aves	46	30	241	29	49	5	421
Total Aves	62	35	256	31	63	5	436

Supplementary Table 10. Herpetological taxa from late Middle and Early Upper Paleolithic levels at Lapa do Picareiro.

	BB	CC	DD	EE	FF	JJ
**Anura**						
Rana perezi						
Rana iberica			3	3		2
Ranidae						2
Bufo bufo	26	9	44	10	1	89
Bufo calamita	3	3	10	1		5
Bufonidae	4		3	1		8
Pelobates cultripes			2			4
**Testudines**						
Testudinidae						1
**Squamata**						
Lacerta lepida						6
Squamata						1
Total indet	101	104	655	108	104	1,993
Total	134	116	717	123	105	2,111
